# Vitamin A administration at birth promotes calf growth and intramuscular fat development in Angus beef cattle

**DOI:** 10.1186/s40104-018-0268-7

**Published:** 2018-07-23

**Authors:** Corrine L. Harris, Bo Wang, Jeneane M. Deavila, Jan R. Busboom, Martin Maquivar, Steven M. Parish, Brent McCann, Mark L. Nelson, Min Du

**Affiliations:** 1 Department of Animal Sciences, Washington State University, Pullman, WA 99164 USA; 2 State Key Laboratory of Animal Nutrition, College of Animal Science and Technology, China Agricultural University, Beijing, 100193 People’s Republic of China; 3 College of Veterinary Medicine, Washington State University, Pullman, WA 99164 USA

**Keywords:** Beef, Calf, Cattle, Marbling fat, Quality, Vitamin A

## Abstract

**Background:**

Marbling, or intramuscular fat, is an important factor contributing to the palatability of beef. Vitamin A, through its active metabolite, retinoic acid, promotes the formation of new fat cells (adipogenesis). As intramuscular adipogenesis is active during the neonatal stage, we hypothesized that vitamin A administration during the neonatal stage would enhance intramuscular adipogenesis and marbling.

**Methods:**

Angus steer calves (*n* = 30), in a completely randomized design, were randomly allotted to three treatment groups at birth, receiving 0, 150,000, or 300,000 IU of vitamin A at both birth and one month of age. A biopsy of the *biceps femoris* muscle was collected at two months of age. After weaning at 210 d of age, steers were fed a backgrounding diet in a feedlot until 308 d of age, when they were transitioned to a high concentrate finishing diet and implanted with trenbolone/estradiol/tylosin mixture. Steers were harvested at an average of 438 d of age. All diets were formulated to meet nutrient requirements.

**Results:**

Weaning weight and weight during the backgrounding phase were linearly increased (*P* <  0.05) by vitamin A level, though no difference in body weight was observed at harvest. Intramuscular fat of steers at 308 d of age, measured by ultrasound, quadratically increased (*P* <  0.05) with vitamin A level from 4.0±0.26 % to 4.9±0.26 %. Similarly, carcass marbling score in the ribeye quadratically increased (*P* < 0.05).

**Conclusion:**

Administration of vitamin A at birth increased weaning weight and enhanced marbling fat development. Thus, vitamin A administration provides a practical method for increasing marbling and early growth of beef cattle.

## Background

As the major contributor to beef palatability, marbling, or intramuscular fat deficiency poses a major problem to both beef producers and beef consumers [1]. Marbling was identified as one of the top beef quality problems according to surveys of beef producers by the National Cattlemen’s Beef Association [2–4].

Marbling fat can be increased through both intramuscular adipocyte hyperplasia and hypertrophy, of which adipocyte hyperplasia is especially important because these adipocytes provide sites for later lipid deposition. Adipocytes are primarily developed during the fetal and neonatal stages [5, 6]. Fibro/adipogenic progenitor cells (FAPs) in the stromal-vascular fraction of skeletal muscle serve as the primary reservoir of intramuscular adipocytes [7–9]. Retinoic acid, a primary metabolite of vitamin A, is a ligand for retinoic acid receptor (RAR) and retinoid X receptor (RXR). In mice, we observed that maternal vitamin A promotes angiogenesis during fetal adipose tissue development, which increases the density of adipogenic progenitor cells mediated by RAR signaling [10]. Though new adipocytes can be generated throughout the life of animal, the major stage of adipocyte formation occurs during fetal and neonatal stages, with intramuscular adipogenesis occurring later compared to other fat depots [11–13]. Consequently, we hypothesized that injecting vitamin A during the neonatal stage would promote intramuscular adipocyte formation, providing sites for later marbling fat accumulation.

In rodents, we also found that maternal dietary vitamin A increases the growth of offspring mice [10]. Retinoic acid can increase myogenic progenitor pool in human embryonic stem cells and myogenesis in zebra fish [14, 15]. Because skeletal muscle composes a large portion of beef carcasses, we also hypothesized that neonatal vitamin A supplementation would increase muscle development and therefore growth efficiency of steers.

## Methods

### Animals

A total of thirty Black Angus (*Bos taurus primigenius*) steer calves were randomly selected during spring calving from an Angus based population of cows and heifers at the Washington State University Ensminger Beef Center. Cows and heifers had been synchronized using a 5-d CO-Synch + CIDR protocol. On d 0 of the protocol, cows were injected with gonadotropin-releasing hormone (GnRH) and a controlled internal drug release (CIDR) unit was placed. After 5 d, the CIDR was removed and animals were injected with prostaglandin F_2α_ (PGF_2α_). Cows were injected 6 ± 2 h later with a second shot of PGF_2α_ and artificially inseminated by fixed time AI 3 d after the CIDR was removed [16]. Calves were allotted among treatments, within each set of 3 bull calves by birth order, to randomly distribute age (average age was the same for all three treatments; within treatments, the age range was ±6 d). There were 9 Angus sires represented in calves, which were distributed across treatments. No twin male calves were included in the study. Within 24 h of birth, calves were castrated, vaccinated (Alpha7 Cattle Vaccine, Boehringer Ingelheim, Ridgefield, CT) and given injections (i.m.) in the “injection triangle” of the neck based on their selected treatment group. Control animals received a placebo injection of glycerol (*n* = 10), while treatment animals received either 150,000 international units (IU) (*n* = 10) or 300,000 IU (n = 10) of vitamin A (200,000 IU/mL Retinol-palmitate in glycerol, Stuart Products, Bedford, TX 76022) at birth and at 1 month of age. Cow-calf pairs were managed together, grazing native and improved pasture. All calves were fence-line weaned at an average of 210 ± 6 d of age and grazed improved pasture until transported to the Washington State University Cattle Feeding Laboratory at an average of 225 d.

After weaning, steers were blocked by weight and vitamin A treatment group into 6 feedlot pens with steers of each treatment into two pens. Animals were offered feed and water ad libitum for an average of 213 d on feed, with calves fed a backgrounding diet (50% steam-rolled corn, 30% grass hay, 15% potato co-products and 5% dry supplement) for 84 d with free-choice trace mineral salt (98% NaCl, 0.509% Se, 0.006% Co, 0.01% I, 0.035% Cu, 0.20% Fe, 0.18% Mn, 0.037% Mg, 0.35% Zn). The backgrounding diet was formulated to contain 12% crude protein (CP), 21% neutral detergent fiber (NDF), 3% ether extract (EE), 70% total digestible nutrients (TDN), 1.8 Mcal NEm/kg DM and 1.1 Mcal NEg kg DM. All diets were formulated to meet the National Academies of Sciences, Engineering and Medicine [17] nutrient requirements for beef cattle. Three step-up diets were fed for 7 d each and increased concentrate by equal percentage. The final finisher was comprised of 59.5% steam rolled corn, 24% potato co-products (20% potato pieces, 4% cooked French fries), 8% grass hay, 5% dry supplement and 3.5% yellow grease. Nutrient analysis of the grass hay and final finisher can be found in Table 1, and the composition of the dry supplement is listed in Table 2. Diet samples were collected as a weekly composite and analyzed by near-infrared spectroscopy (NIR). At an average of 308 d of age, steers were implanted with Component TE-IS with Tylan (Elanco, Greenfield IL). All animals were weighed monthly. Feed intake was calculated using a pen as an experimental unit by recording feed consumption, which was then divided by animal-days in each pen to obtain feed consumption per animal.

**Table 1:** Nutrient analysis of grass hay and final finisher on DM basis

Component	Grass hay	Final finisher
Crude protein, %	6.4	13.1
Available protein, %	5.8	13.1
ADF^a^, %	35.2	7.2
aNDF^b^, %	56.5	11.4
Lignin, %	4.8	2.0
NFC^c^, %	28.7	63.3
Starch, %	1.1	55.1
Crude fat, %	2.3	6.6
Ash, %	6.08	5.60
TDN^d^, %	61	85
NEm, Mcal/lb.	0.57	0.98
NEg, Mcal/lb.	0.32	0.67
Ca, %	0.27	--
P, %	0.11	--

^a^ADF: Acid detergent fiber

^b^aNDF: Amylase-treated neutral detergent fiber

^c^NFC: Nonfiber carbohydrates

^d^TDN: Total digestible nutrients

**Table 2:** Composition of the dry supplement

Nutrient	Amount (DM)
Crude protein	76.9%
Crude fat	1.9%
Ca	12.31%
P	1.03%
Na	4.09%
K	0.3%
Mg	2.08%
S	0.24%
Mn	615 ppm
Zn	924 ppm
Cu	308 ppm
Co	26 ppm
I	87 ppm
Se	6–8 ppm
Vitamin A	90,200 IU/kg
Vitamin D	9020 IU/kg
Vitamin E	226 IU/kg
Rumensin® 0.68 g/kg	616 g/t
Tylan ® 0.14 g/kg	123 g/t

Steers were harvested at the Washington State University Meats Laboratory at either 426 (2 steers of each group), 433 (2 control, 1,150,000 IU, 3,300,000 IU steers), 440 (2 steers of each group), or 454 d (3 control, 2,150,000 IU, 2,300,000 IU steers) of age based on body weight and balanced for days on feed among treatments. In order to calculate average daily gain, the following equation was used: [(weight at Cattle Feeding Laboratory night prior to slaughter corrected for rumen fill – initial weight)/days on feed]. Average age at slaughter was 438 d.

### Collection and analysis of muscle biopsy and serum

At 60 ± 6 d of age (varied due to birth order), blood was collected from the steer calves by a tail vein punctuation, and serum was separated and stored in dark at − 80°C to prevent isomerization and degradation of retinoids. Once retinoids were extracted according to a published protocol [18], contents were measured by high performance liquid chromatography (HPLC) using a reverse phase column (Luna 3 μm C18(2) 100 Å, LC Column 150 mm × 3 mm, Phenomenex, Torrance, CA). The mobile phase (methanol 65%/H_2_O 35%) was pumped at 1.0 mL/min and retinol, retinoic acid and retinaldehyde were detected at a wavelength of 325, 340 and 385 nm, respectively. Standards (Sigma-Aldrich, St. Louis, MO) were used for calculating standard curves and recovery rates [18].

A biopsy of the *biceps femoris* from the hind right leg was taken from each calf at 2 months of age. A small area (12 cm × 12 cm) was clipped and prepped surgically with a combination of povidine iodine scrub, 75% ethanol and povidine solution. Once 2% lidocaine was injected as an anesthetic, a surgical blade was used to make a 2-cm incision through the skin to collect approximately 1 to 2 g of skeletal muscle tissue with blunt-end scissors. The skin incision was closed by stitching, and a portion of the sample was frozen in liquid nitrogen and stored at − 80°C until analyzed.

The biopsy muscle was separated into two portions, with a small portion used for paraffin imbedding for structural analyses, and the large portion frozen in liquid nitrogen for later analyzing Zinc finger protein 423 (*ZFP423*) and peroxisome proliferator-activated receptor γ (*PPARG*) expression as previously described [19]. Briefly, total RNA was extracted with TRIzol reagent, followed by DNase (NEB, Ipswich, MA) treatment to remove DNA. An iScriptTM cDNA synthesis kit (Bio-Rad, Hercules, CA) was used to synthesize cDNA. Quantitative Real-Time PCR (q-RT-PCR) was performed using a CFX RT-PCR detection system (Bio-Rad) with a SYBR green RT-PCR kit from Bio-Rad. The mRNA expression of three enzymes involved in retinoid metabolism, including alcohol dehydrogenase (*ADH*) 4, *ADH5*, and aldehyde dehydrogenase 1 A1 (*ALDH1A1*) were also measured. Primer Sequences can be found in Table 3. All PCR efficiencies were above 96%, with the cycling conditions comprised at 2 min polymerase reaction (95°C) and then 40 cycles at 95°C for 15 s and 60°C for 30 s. Relative mRNA expression was determined after normalization to 18S rRNA using the –ΔΔCt method [19]. Glyceraldehyde 3 phosphate dehydrogenase (*GAPDH*) and *β-catenin* were used to verify the validity of 18S rRNA as a reference gene, all of which were unaffected by vitamin treatment. All gene expressions were presented as fold changes to that of the control group.

**Table 3:** Primer sequences for RT-qPCR

Gene	Forward primer sequence (5′→3′)	Reverse primer sequence (5′→3′)	Access number	Product length, bp
*ZFP423*	GGATTCCTCCGTGACAGCA	TCGTCCTCATTCCTCTCCTCT	NM_001101893.1	120
*PPARG*	AGCTCCAAGAATACCAAAGTGCGAT	AGGTTCTTCATGAGGCCTGTTGTAGA	NM_181024.2	98
*ADH4*	AGAAAATGGGCACCAAGGGAA	TTCATTGCTGAGGGGCTTGT	NM_001102059.2	80
*ADH5*	TTGAGATGACTGACGGGGGA	CTCCCACTACCACGCTGATG	NM_001034249.2	117
*ALDH1A1*	GGAAATGGTCGAGAACTGGGA	GGAGGGCTTGGGTGTCATAG	NM_174239.2	299
*GAPDH*	TGCCCGTTCGACAGATAGCC	GCGACGATGTCCACTTTGCC	NM_001034034.2	148
*β−atenin*	CGGCTTTCGGTTGAGCTGAC	GCCGTACCCACCAGAGTGAA	XM_005222525.3	159
18S rRNA	CCTGCGGCTTAATTTGACTC	AACTAAGAACGGCCATGCAC	NR_036642.1	118

### Ultrasound

Steers were subjected to ultrasound at an average of 308 d of age prior to being transitioned from the background to the finishing diet and evaluated for rump fat (cm), fat over the 12^th^ rib (cm), rib eye area (REA, square cm), and amount of intramuscular fat (percent). The ultrasound was completed by a certified technician using an Aquila ultrasound machine (Esaote Pie Medical, Indianapolis, IN) and analyzed using Centralized Ultrasound Processing (CUP) software (https://www.cuplab.com/).

### Collection of carcass data

On the day of harvest, hot carcass weight (HCW) and harvest order was recorded. Carcasses were halved and aged at 4°C for 14 d. Then, the right side of the carcass was ribbed in between the 12^th^ and 13^th^ rib and backfat thickness, REA, kidney, pelvic and heart (KPH) fat, marbling and carcass grading were measured according to U.S. Department of Agriculture standard methods by Dr. Busboom in a blind fashion [20]. For USDA quality grading, numerical marbling scores are assigned; 500 is the minimum score for a small amount of marbling (USDA Choice), 600 for a modest amount and 700 for a moderate amount. A 2.5-cm ribeye steak was collected from the right side of the carcass at time of ribbing, wrapped and immediately stored in the freezer (− 30°C) until analysis. Yield grade was calculated [2.5 + (6.35 × adjusted fat thickness, inches) + (0.2 × % KPH) + (0.0038 × HCW, pounds) – (0.32 × REA, square inches) for each individual steer [21]. Dressing percent was calculated by dividing HCW by final live body weight using a 4% shrink.

### Ribeye preparation

Ribeye steaks were analyzed for cooking loss and slice shear force (SSF). Steaks were thawed at 4°C for 48 h, weighed and then cooked on a preheated grill (George Foreman Double-Sided Grill, Beachwood, OH). Temperature of the geometric center was monitored by a 12 Channel Scanning Thermocouple Thermometer (Model 692–8010, Barnart, Barrington, IL). Steaks were removed from the grill upon reaching 71°C. Cooked steaks were weighed and prepared for SSF measurement. Cooking loss was calculated by ([(weight before cooking – weight after cooking) / weight before cooking] × 100). A 2.5-cm thick, 5-cm long slice parallel to the muscle fibers was removed from the lateral end of each steak and cooled to room temperature (21°C). A Warner-Bratzler Meat Shear Mecmesin (G-R Manufacturing Co., Manhattan, KS) fitted with a flat blade designed for SSF was used to measure the samples [22].

### Nitrogen, crude protein and fat analysis

Samples from the anterior end of the rib-eye were collected, freeze-dried and ground to 1 mm particle size using a table top grinder (Hamilton-Beach Fresh-Grind Coffee Grinder, Glen Allen, VA). Total crude fat was determined by the Mojonnier Ether Extraction system (AOAC# 954.02, Weber Scientific, Hamilton, NJ). The samples were also analyzed for total nitrogen (N) by combustion in a CN628 Carbon/Nitrogen Determinator (AOAC# 992.15, Leco, St. Joseph, MI). When the sample was combusted, N release was measured and converted to crude protein (CP) (CP = N × 6.25).

### Statistical analysis

The experiment was designed as a completely randomized design (CRD) with a one-way treatment structure. All data except for animal performance data were analyzed using the Mixed procedure of SAS (SAS Inst. Inc., Cary, NC) with animal as the experimental unit. Treatment was three levels of injection: control (0 IU), low-level (150,000 IU) and high-level (300,000 IU). To account for pen effects, dry matter intake (DMI), average daily gain (ADG) and feed to gain ratio (F/G, feed conversion rate) were analyzed using the Mixed procedure of SAS in a randomized complete block design, with pen as the experimental unit. In addition, One-way ANOVA followed by a post hoc Tukey’s test was used to compare difference between treatments by treating level of vitamin A as a discrete variable. Originally, each treatment level had the same sample size (*n* = 10). Four steers were removed from the experiment prior to weaning (one in the control treatment, three in the 150,000 IU treatment) due to respiratory disease. A steer from the 300,000 IU treatment bloated after 99 d being fed the finishing diet. As a result, *n* per treatment differs between performance measures with *n* = 9 for control, *n = *7 for 150,000 IU and *n = *9 for 300,000 IU vitamin A treatments. All collected samples are included in the data analysis.

## Results

### Animal performance

A summary of means (± SE) and *P*-values for response variables are included in Table 4. Data are arranged temporally. As expected, birth weight did not differ among treatments. Weaning weight linearly increased (*P* = 0.0178) with vitamin A treatment. Cattle that received an injection of 150,000 IU or 300,000 IU of vitamin A exhibited greater weaning weights, but did not differ from each other. As a result, the ADG was greater in vitamin A treated calves compared to control calves. Consistently, at the transition to finishing phase (309 d), the weights of vitamin A treated groups were higher than control group. However, the weight difference disappeared at the end of the finishing phase (438 d of age), partially due to the increasing variation among individual animals within each treatment (Table 4).

**Table 4:** Impacts of neonatal vitamin A administration on cattle growth performance

Response	0 IU (*n* = 9)	150,000 IU (*n* = 7)	300,000 IU (*n* = 9)	SE	*P*-value
Birth to weaning					
Birth weight, kg	35.1	35.6	35.6	0.58	0.932
Average daily gain, kg/d	0.88^b^	0.98^a^	1.00^a^	0.02	0.034
Backgrounding					
Weaning weight at 210 d, kg	219.2^b^	248.7^ab^	246.0^a^	5.98	0.018
Dry matter intake, kg/(head·d)	9.09	8.89	9.25	0.39	0.320
Average daily gain, kg	1.35	1.37	1.47	0.17	0.784
Gain: Feed ratio, kg	0.149	0.153	0.156	0.031	0.944
Finishing					
Weight at 308 d, kg	312.1^b^	333.0^ab^	339.7^a^	8.65	0.040
Dry matter intake, kg/(head·d)	8.95	9.45	9.09	0.85	0.829
Average daily gain, kg	2.06	1.99	1.90	0.17	0.627
Feed: Gain ratio, kg	4.35	4.76	4.79	0.13	0.219
Pre-harvest					
Actual body weight (438 d), kg	581.8	610.4	588.3	10.97	0.116

^a-b^Means in the same row with different letter differ significantly

Vitamin A had no effects (*P >* 0.05) on animal performance during the backgrounding or finishing phase. Backgrounding DMI, ADG and F/G did not differ between vitamin A treatment, similarly to finishing DMI, ADG and F/G. Hip height was also measured when cattle were weighed and was not significantly different among treatment groups (data not shown).

### Vitamin A and ZFP423 measurement at 2 months of age

**Fig. 1:**
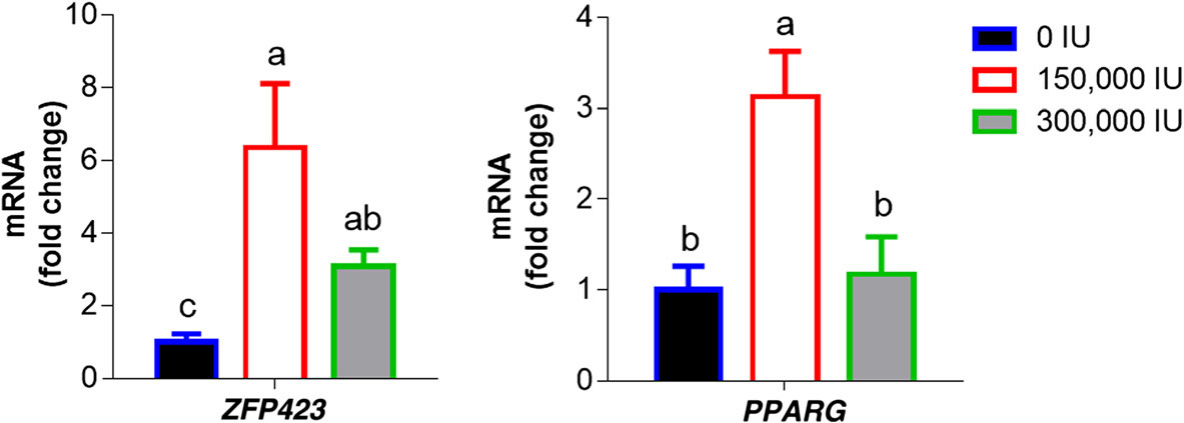
*ZFP423* and *PPARG* mRNA expression in *biceps femoris* muscle samples of calves at 2 months of age. One-way ANOVA for Complete Randomized Design. Means ± SE. ^a-c^ Bars in the same gene with different letters differ significantly (*P* < 0.05). (0 IU, *n* = 9; 150,000 IU, *n* = 7; 300,000 IU, *n* = 9)

Three vitamin A metabolites in plasma were measured: retinoic acid, retinaldehyde and retinol (Table 5). At 2 months of age, there was no effect of vitamin A injection on plasma retinaldehyde. The largest difference (*P* = 0.0001) was seen in concentration of retinoic acid with the 150,000 IU vitamin A group having the highest content of retinoic acid. Relative mRNA expression of *ZFP423* and *PPARG*, two key transcription factors regulating adipogenesis, was also significantly higher in the low-level vitamin A treatment (Fig. 1), which was consistent with the higher level of retinoic acid in the 150,000 IU vitamin A group, showing an association between retinoic acid levels and *ZFP423* expression.

**Table 5:** Serum retinoid concentrations and mRNA expression of genes involved in retinoid metabolism in intramuscular fat of 2 months old of calves receiving different doses of vitamin A treatments

Response	0 IU (*n* = 9)	150,000 IU (*n* = 7)	300,000 IU (*n* = 9)	SE	*P*-value
Serum retinoid content					
Retinoic acid, pmol/mL	7.05^b^	13.16^a^	11.02^a^	0.81	< 0.001
Retinaldehyde, pmol/mL	10.51	13.26	11.71	1.28	0.348
Retinol, nmol/mL	1.14^ab^	1.21^a^	0.94^b^	0.06	0.026
mRNA abundance of enzymes (Arbitrary unit)					
*ADH4*, relative expression	1.00	0.68	0.35	0.14	0.184
*ADH5*, relative expression	1.00	2.01	1.82	0.21	0.123
*ALDH1A1*, relative expression	1.00	1.71	1.35	0.20	0.369

^a-b^Means in the same row with different letters differ significantly (*P* < 0.05)

To discern the possible reasons for the higher retinoic acid level in the 150,000 IU vitamin A group, the mRNA expression of three most significant enzyme contributors to retinoid metabolism was measured at 2 months of age: *ADH4*, *ADH5* and *ALDH1A1*. Linear trends (*P <* 0.10), which are not considered to be statistically significant, were seen in both *ADH4* and *ADH5* expression, but *ADH4* decreased with vitamin A treatment, while *ADH5* increased with vitamin A treatment (Table 5). There were no effects of vitamin A on *ALDH1A1* expression.

### Ultrasound data

There was a quadratic increase (*P <* 0.05) in intramuscular fat (IMF) measured by ultrasound at an average of 308 d of age with IMF highest in the 150,000 IU vitamin A group (Table 6). Body weight was a significant covariate but there was no interaction (*P* > 0.05) between body weight and IMF. The higher IMF in the low-level indicates that *ZFP423* and *PPARG* expression was correlated to intramuscular fat deposition (Fig. 1). Treatment had no effects (*P* > 0.05) on rib fat, rump fat and REA, though the 300,000 IU had a trend of increase when compared to the control group (*P* = 0.07).

**Table 6:** Ultrasound measurement, carcass composition and meat quality of steers received different doses of vitamin A treatments at birth

Response	0 IU (*n* = 9)	150,000 IU (*n* = 7)	300,000 IU (*n* = 9)	SE	*P*-value
Ultrasound at transition to the finishing stage					
IMF, %	3.95^b^	4.93^a^	4.34^ab^	0.26	0.036
Rib fat, cm	0.38	0.38	0.41	0.03	0.659
Rump fat, cm	0.53	0.53	0.43	0.05	0.240
REA, cm^2^	53.5	56.1	58.3	2.21	0.337
Carcass characteristics					
Carcass weight, kg	341.0	357.0	349.5	3.87	0.527
Dressing percent, %	59.5	59.1	59.8	0.29	0.468
Marbling score^*^	583.3^b^	671.7^a^	610.0^ab^	20.20	0.016
KPH, %	2.34	2.18	2.15	0.14	0.661
REA, cm^2^	82.2	85.6	84.2	3.87	0.565
Back fat, cm	1.29	1.46	1.27	0.06	0.421
Yield grade	3.02	3.16	2.96	0.09	0.921
Meat quality					
Cooking loss, %	20.3	20.6	21.5	1.28	0.793
SSF, kg	8.45	10.27	9.66	1.12	0.637
Meat composition					
CP%	23.4	23.3	23.9	0.38	0.438
IMF%	6.33	7.49	6.94	0.69	0.572

^*^500 = small 0; 600 = modest 0; 700 = moderate 0

^a-b^Means in the same row with different letters differ significantly (*P* < 0.05)

### Carcass characteristics

Injection of vitamin A had no effect (*P* > 0.05) on carcass weight, dressing percent, KPH, REA, backfat or yield grade (Table 6). Marbling score increased quadratically (*P <* 0.02) with vitamin A. Marbling score was the highest (*P* < 0.05) in the 150,000 IU vitamin A treatment and consistent with IMF measured at about 308 d of age through ultrasound of the cattle.

### Meat quality data

There were no effects of vitamin A on cooking loss, SSF, crude protein content or fat content (Table 6). These data show that neonatal vitamin A treatment had no significant effect on meat quality characteristics with the exception of marbling.

## Discussion

Eating quality of beef is determined by juiciness, tenderness and flavor [23]. Marbling, or intramuscular fat deposition, is highly related to meat quality and a positive eating experience [24, 25]. In the United States, the primary system used for beef evaluation is the United States Department of Agriculture beef grading system, and amount of marbling is one of the major factor in determining the official quality grade of young beef carcasses (Prime, Choice, Select, Standard). Similar beef grading systems also exist in other countries. Thus, great efforts have been exerted by beef cattle industry to enhance marbling [2].

In ruminant animals, adipogenesis begins around mid-gestation, overlapping the period of major muscle fiber formation between 2 and 7 months of gestation [6]. Muscle fibers, intramuscular adipocytes and fibroblasts are derived from a common population of mesenchymal progenitor cells during early embryonic development, and the commitment to the myogenic or fibro/adipogenic cell lineage occurs during this period [26, 27]. Manipulation of adipogenesis during the stage critical for intramuscular adipocyte formation is expected to enhance marbling [9].

Through comparing adipogenic and non-adipogenic rodent fibroblasts, ZFP423 has been identified as a critical regulator for initial stage of adipogenesis, which further coordinates with PPARG to regulate late stage adipogenesis in mice [28, 29]. In a later study, ZFP423 has been demonstrated to regulate adipogenesis of bovine stromal vascular cells [30]. Retinoic acid, a metabolite of vitamin A, is essential for vertebrate organogenesis [31], including adipose tissue development [32]. As we recently reported, maternal retinoid supplementation increases adipose progenitor pool in offspring [10]. Because adipose progenitor cells express *ZFP423*, the higher *ZFP423* expression and intramuscular fat content in vitamin A treated calves were likely due to an increased population of adipose progenitors. The recent discoveries regarding FAPs, ZFP423 and retinoic acid have yielded the concept of a nutrigenomic approach for the manipulation of progenitor cells inside skeletal muscle to increase marbling [33].

While vitamin A (retinol-palmitate) administration clearly increased expression of *ZFP423* in both the low-level and high-level vitamin A group, the increase was highest in the low-level treatment group, which also demonstrated the highest concentration of serum retinoic acid. However, the decrease in serum retinoic acid concentration in the high-level treatment group compared to the low-level treatment was surprising, as it was presumed that a larger injection dose of vitamin A would result in higher levels of retinoic acid, thus further up-regulating *ZFP423* expression. To provide a possible explanation, we measured the mRNA expression of enzymes governing retinoid metabolism, because transcriptional regulation mainly controls their enzymatic activities [34]. Retinol is converted into retinaldehyde by ADH and retinol dehydrogenases (RDH), while retinoic acid is generated from retinaldehyde by aldehyde dehydrogenases (ALDH) [35]. However, no significant differences were found in enzyme expression. Thus, it seems unlikely that enzyme expression was related to the observed retinoid differences, as the trends for enzyme expression were linear while retinoid content exhibited significant quadratic increases.

When vitamin A was injected in high doses to rats, it was demonstrated that high doses lead to a proportionally higher amount of the injected dose being stored immediately in the liver [36]. Thus, it was possible that the high-level group would have had less vitamin A overall available for metabolism at one month after the second vitamin A injection due to the increased liver storage, providing a possible explanation for the lower retinoic acid concentration in the higher vitamin A group compared to the lower group. Additionally, the higher dose of vitamin A administration could accelerate retinoid catabolism and clearance, which resulted in lower retinoic acid concentration one month following vitamin A injection, an “overcompensation” phenomenon [37].

The increase in IMF at an average of 308 d of age and marbling in the harvested cattle treated with 150,000 IU of vitamin A was consistent with *ZFP423* and *PPARG* expression and validates our proposed approach to increasing marbling in beef cattle. While there were differences in marbling score, there was no significant effect of vitamin A on total crude fat in the ribeye. The lack of difference in crude fat contents in ribeye muscle could be due to three reasons: 1) the sample sizes were too small and additional animals might be needed to provide sufficient power to discern the crude fat difference; 2) only a small portion of the ribeye muscle was used for chemical measurement, which increased variation; and/or 3) a potential reduction in fat content of muscle fibers due to vitamin A treatment which enhanced muscle oxidation capacity. Furthermore, it is possible that the high vitamin A treatment increased the density of preadipocytes and adipocytes, but these immature adipocytes had insufficient accumulation of lipid due to the harvest at a younger age (438 d) compared to most cattle harvested commercially. Another possibility could be due to enhanced lipid oxidation in adipocytes. Retinoic acid can induce lipid oxidation in mature adipocytes, which limits lipid accumulation [33]. Consistently, vitamin A deficiency is frequently used to enhance marbling fat accumulation in feedlot cattle [38–42]. In the absence of vitamin A, lipid oxidation in adipocytes reduces; as a result, intramuscular adipocytes accumulate more lipids, resulting in adipocyte hypertrophy and increased marbling fat deposition.

We did not observe difference in meat quality among three treatments. Due to the small, yet significant, changes in marbling score but not in percentage of crude fat, it was not surprising to find that other meat characteristics were the same between all vitamin A treatments. Of note, in this study, we examined *biceps femoris* biopsy of steer calves at 2 months and *longissimus* muscle of steers at harvest. Though we expect overall changes in intramuscular adipogenesis of different muscles to be very similar, the difference in muscle composition might partially explain the lack of difference in meat quality at harvest.

In this study, we found that vitamin A administration significantly enhanced the growth rate of calves, which was consistent with our earlier observation in mice, where maternal vitamin A supplementation enhanced the growth rate of offspring [10], while retinoic acid deficiency reduced growth [34]. Greater weaning weight is promising for beef producers, especially cow-calf producers, as it represents a significant calf price increase. A heavier weaned calf may also require less days on feed to reach a finishing weight, making vitamin A a potential target for increasing the efficiency of beef production. The response in weaning weight could also suggest an as-of-yet undefined role of vitamin A in muscle development. Because no hip height difference was observed among treatments, the increase in weaning weight suggested possible increase in muscle growth, which was consistent with the observed trend of increase in REA in vitamin A treated steers. Mechanisms leading to increased weaning weight in vitamin A treated groups need to be further examined. However, the weight difference did not reach significance at harvest, which could be due to increased variation among individual animals as cattle grew, rendering our study underpowered to discern treatment effects at harvest. Alternatively, it could also suggest that the effect of neonatal vitamin A administration on calf growth wanes as calf age increases, suggesting recurring vitamin A administration beyond the neonatal stage may be needed.

## Conclusion

Marbling is a vital component of high quality beef, partially determining tenderness and palatability. Western culture has developed a taste preference for juicy, grain-fed beef and marbling is necessary to achieve this standard. Aside from this taste preference, beef cattle producers can find significant profit increases associated with increased marbling. Vitamin A successfully increased IMF in cattle prior to the switch to a finishing diet and improved marbling scores at harvest, which has large financial rewards for beef producers. Injection of vitamin A to neonatal calves successfully increased expression of *ZFP423*. Previous studies have demonstrated vitamin A increased adipogenic progenitor density, which explains the increase in *ZFP423* expression and may be responsible for the observed increase in intramuscular fat. The increase in weaning weight could also suggest that vitamin A plays a role in muscle development or animal growth. Thus, vitamin A administration during early development potentially provides beef cattle producers with a practical method to enhance marbling and increase the efficiency of beef production.

## Abbreviations

ADH: Alcohol dehydrogenase
ALDH1A1: Aldehyde dehydrogenase-1 A1
CIDR: Controlled internal drug release
FAP: Fibro/adipogenic progenitor cell
GAPDH: Glyceraldehyde 3-phosphate dehydrogenase
NIR: Near-infrared spectroscopy
PPARG: Peroxisome proliferator-activated receptor
RAR: Retinoic acid receptor
RDH: Retinol dehydrogenases
RXR: Retinoid X receptor
ZFP423: Zinc finger protein 423

## References

[1] Moore MC, Gray GD, Hale DS, Kerth CR, Griffin DB, Savell JW (2012). National Beef Quality Audit-2011: in-plant survey of targeted carcass characteristics related to quality, quantity, value, and marketing of fed steers and heifers. J Anim Sci.

[2] Boykin CA, Eastwood LC, Harris MK, Hale DS, Kerth CR, Griffin DB (2017). National Beef Quality Audit-2016: in-plant survey of carcass characteristics related to quality, quantity, and value of fed steers and heifers. J Anim Sci.

[3] Garcia LG, Nicholson KL, Hoffman TW, Lawrence TE, Hale DS, Griffin DB (2008). National Beef Quality Audit-2005: survey of targeted cattle and carcass characteristics related to quality, quantity, and value of fed steers and heifers. J Anim Sci.

[4] Igo JL, VanOverbeke DL, Woerner DR, Tatum JD, Pendell DL, Vedral LL (2013). Phase I of the National Beef Quality Audit-2011: quantifying willingness-to-pay, best-worst scaling, and current status of quality characteristics in different beef industry marketing sectors. J Anim Sci.

[5] Hausman GJ, Dodson MV, Ajuwon K, Azain M, Barnes KM, Guan LL (2009). Board-invited review: the biology and regulation of preadipocytes and adipoc ytes in meat animals. J Anim Sci.

[6] Du M, Tong J, Zhao J, Underwood KR, Zhu M, Ford SP (2010). Fetal programming of skeletal muscle development in ruminant animals. J Anim Sci.

[7] Uezumi A, Fukada S, Yamamoto N, Takeda S, Tsuchida K (2010). Mesenchymal progenitors distinct from satellite cells contribute to ectopic fat cell formation in skeletal muscle. Nature Cell Biol.

[8] Hausman GJ, Poulos S (2004). Recruitment and differentiation of intramuscular preadipocytes in stromal-vascular cell cultures derived from neonatal pig semitendinosus muscles. J Anim Sci.

[9] Du M, Huang Y, Das AK, Yang Q, Duarte MS, Dodson MV (2013). MEAT SCIENCE AND MUSCLE BIOLOGY SYMPOSIUM: manipulating mesenchymal progenitor cell differentiation to optimize performance and carcass value of beef cattle. J Anim Sci.

[10] Wang B, Fu X, Liang X, Wang Z, Yang Q, Zou T (2017). Maternal Retinoids increase PDGFRalpha+ progenitor population and beige Adipogenesis in progeny by stimulating vascular development. EBioMedicine.

[11] Cianzio DS, Topel DG, Whitehurst GB, Beitz DC, Self HL (1985). Adipose tissue growth and cellularity: changes in bovine adipocyte size and number. J Anim Sci.

[12] Robelin J (1981). Cellularity of bovine adipose tissues: developmental changes from 15 to 65 percent mature weight. J Lipid Res.

[13] Taga H, Bonnet M, Picard B, Zingaretti MC, Cassar-Malek I, Cinti S (2011). Adipocyte metabolism and cellularity are related to differences in adipose tissue maturity between Holstein and Charolais or blond d’Aquitaine fetuses. J Anim Sci.

[14] Ryan T, Liu J, Chu A, Wang L, Blais A, Skerjanc IS (2012). Retinoic acid enhances skeletal myogenesis in human embryonic stem cells by expanding the premyogenic progenitor population. Stem Cell Rev.

[15] Hamade A, Deries M, Begemann G, Bally-Cuif L, Genet C, Sabatier F (2006). Retinoic acid activates myogenesis in vivo through Fgf8 signalling. Dev Biol.

[16] Stevenson JS, Thompson KE, Forbes WL, Lamb GC, Grieger DM, Corah LR (2000). Synchronizing estrus and(or) ovulation in beef cows after combinations of GnRH, norgestomet, and prostaglandin F2alpha with or without timed insemination. J Anim Sci.

[17] NASEM (2016). Nutrient requirements of beef cattle. Eighth revised edition.

[18] Kim YK, Quadro L (2010). Reverse-phase high-performance liquid chromatography (HPLC) analysis of retinol and retinyl esters in mouse serum and tissues. Methods Mol Biol.

[19] Duarte MS, Paulino PV, Das AK, Wei S, Serao NV, Fu X (2013). Enhancement of adipogenesis and fibrogenesis in skeletal muscle of wagyu compared with Angus cattle. J Anim Sci.

[20] Underwood KR, Tong JF, Price PL, Roberts AJ, Grings EE, Hess BW, Means WJ, Du M (2010). Nutrition during mid to late gestation affects growth, adipose tissue deposition, and tenderness in cross-bred beef steers. J Anim Sci.

[21] USDA. United States standards for grades of carcass beef. In: Agriculture USDo, editor. https://www.ams.usda.gov/sites/default/files/media/Carcass%20Beef%20Standard.pdf: Agricultural Marketing Service; 2016.

[22] Wheeler TL, Shackelford SD, Koohmaraie M (2007). Beef longissimus slice shear force measurement among steak locations and institutions. J Anim Sci.

[23] Roeber DL, Cannell RC, Belk KE, Miller RK, Tatum JD, Smith GC (2000). Implant strategies during feeding: impact on carcass grades and consumer acceptability. J Anim Sci.

[24] O'Quinn TG, Brooks JC, Miller MF (2015). Consumer assessment of beef tenderloin steaks from various USDA quality grades at 3 degrees of doneness. J Food Sci.

[25] Wheeler TL, Cundiff LV, Shackelford SD, Koohmaraie M (2004). Characterization of biological types of cattle (cycle VI): carcass, yield, and longissimus palatability traits. J Anim Sci.

[26] Tong J, Zhu MJ, Underwood KR, Hess BW, Ford SP, Du M (2008). AMP-activated protein kinase and adipogenesis in sheep fetal skeletal muscle and 3T3-L1 cells. J Anim Sci.

[27] Zhu MJ, Han B, Tong J, Ma C, Kimzey JM, Underwood KR (2008). AMP-activated protein kinase signalling pathways are down regulated and skeletal muscle development impaired in fetuses of obese, over-nourished sheep. J Physiol-London.

[28] Gupta RK, Mepani RJ, Kleiner S, Lo JC, Khandekar MJ, Cohen P (2012). Zfp423 expression identifies committed preadipocytes and localizes to adipose endothelial and perivascular cells. Cell Metab.

[29] Gupta RK, Arany Z, Seale P, Mepani RJ, Ye L, Conroe HM (2010). Transcriptional control of preadipocyte determination by Zfp423. Nature.

[30] Huang Y, Das AK, Yang QY, Zhu MJ, Du M (2012). Zfp423 promotes adipogenic differentiation of bovine stromal vascular cells. PLoS One.

[31] Duester G (2008). Retinoic acid synthesis and signaling during early organogenesis. Cell.

[32] Dani C, Smith AG, Dessolin S, Leroy P, Staccini L, Villageois P (1997). Differentiation of embryonic stem cells into adipocytes in vitro. J Cell Sci.

[33] Wang B, Yang Q, Harris CL, Nelson ML, Busboom JR, Zhu MJ (2016). Nutrigenomic regulation of adipose tissue development - role of retinoic acid: a review. Meat Sci.

[34] Molotkov A, Fan X, Deltour L, Foglio MH, Martras S, Farres J (2002). Stimulation of retinoic acid production and growth by ubiquitously expressed alcohol dehydrogenase Adh3. Proc Natl Acad Sci U S A.

[35] Reichert B, Yasmeen R, Jeyakumar SM, Yang F, Thomou T, Alder H (2011). Concerted action of aldehyde dehydrogenases influences depot-specific fat formation. Mol Endocrinol.

[36] Guggenheim K, Koch W (1944). A liver storage test for the assessment of vitamin a. Biochem J.

[37] Tagliaferro AR, Levitsky DA (1982). Overcompensation of food intake following brief periods of food restriction. Physiol Behavior.

[38] Pickworth CL, Loerch SC, Fluharty FL (2012). Effects of timing and duration of dietary vitamin a reduction on carcass quality of finishing beef cattle. J Anim Sci.

[39] Ward AK, McKinnon JJ, Hendrick S, Buchanan FC (2012). The impact of vitamin a restriction and ADH1C genotype on marbling in feedlot steers. J Anim Sci.

[40] Pickworth CL, Loerch SC, Fluharty FL (2012). Restriction of vitamin a and D in beef cattle finishing diets on feedlot performance and adipose accretion. J Anim Sci.

[41] Arnett AM, Dikeman ME, Daniel MJ, Olson KC, Jaeger J, Perrett J (2009). Effects of vitamin a supplementation and weaning age on serum and liver retinol concentrations, carcass traits, and lipid composition in market beef cattle. Meat Sci.

[42] Gorocica-Buenfil MA, Fluharty FL, Bohn T, Schwartz SJ, Loerch SC (2007). Effect of low vitamin a diets with high-moisture or dry corn on marbling and adipose tissue fatty acid composition of beef steers. J Anim Sci.

